# Poly (I:C) therapy decreases cerebral ischaemia/reperfusion injury *via*TLR3-mediated prevention of Fas/FADD interaction

**DOI:** 10.1111/jcmm.12456

**Published:** 2014-10-29

**Authors:** Xia Zhang, Tuanzhu Ha, Chen Lu, Fred Lam, Li Liu, John Schweitzer, John Kalbfleisch, Race L Kao, David L Williams, Chuanfu Li

**Affiliations:** a Department of Surgery, Quillen College of Medicine, East Tennessee State UniversityJohnson City, TN, USA; b Pathology Quillen College of Medicine, East Tennessee State UniversityJohnson City, TN, 37614; c Biometry and Medical Computing and the Center for Inflammation, Quillen College of Medicine, East Tennessee State UniversityJohnson City, TN, 37614; d Infectious Disease and Immunity, Quillen College of Medicine, East Tennessee State UniversityJohnson City, TN, 37614; e Department of Geriatrics5, the First Affiliated Hospital of Nanjing Medical UniversityNanjing, 210029, China

**Keywords:** cerebral ischaemia/reperfusion, stroke, TLR3, Poly (I:C), apoptosis, microglial cells

## Abstract

Toll-like receptor (TLR)-mediated signalling plays a role in cerebral ischaemia/reperfusion (I/R) injury. Modulation of TLRs has been reported to protect against cerebral I/R injury. This study examined whether modulation of TLR3 with poly (I:C) will induce protection against cerebral I/R injury. Mice were treated with or without Poly (I:C) (*n* + 8/group) 1 hr prior to cerebral ischaemia (60 min.) followed by reperfusion (24 hrs). Poly (I:C) pre-treatment significantly reduced the infarct volume by 57.2% compared with untreated I/R mice. Therapeutic administration of Poly (I:C), administered 30 min. after cerebral ischaemia, markedly decreased infarct volume by 34.9%. However, Poly (I:C)-induced protection was lost in TLR3 knockout mice. In poly (I:C)-treated mice, there was less neuronal damage in the hippocampus compared with untreated I/R mice. Poly (I:C) treatment induced IRF3 phosphorylation, but it inhibited NF-κB activation in the brain. Poly (I:C) also decreased I/R-induced apoptosis by attenuation of Fas/FasL-mediated apoptotic signalling. In addition, Poly (I:C) treatment decreased microglial cell caspase-3 activity. *In vitro* data showed that Poly (I:C) prevented hypoxia/reoxygenation (H/R)-induced interaction between Fas and FADD as well as caspase-3 and -8 activation in microglial cells. Importantly, Poly (I:C) treatment induced co-association between TLR3 and Fas. Our data suggest that Poly (I:C) decreases in cerebral I/R injury *via*TLR3 which associates with Fas, thereby preventing the interaction of Fas and FADD, as well as microglial cell caspase-3 and -8 activities. We conclude that TLR3 modulation by Poly (I:C) could be a potential approach for protection against ischaemic stroke.

## Introduction

Stroke is the third leading cause of death and the leading cause of long-term disability in the United States. Each year, 795,000 Americans suffer a new or recurrent stroke 1. Approximately, 610,000 of these are initial strokes and 185,000 are recurrent strokes 1. Ischaemic stroke caused by cerebral ischaemia/reperfusion (I/R) injury accounts for ∼83% of all stroke cases 1. At present, there is no effective treatment for cerebral I/R injury. Recent studies have shown that Toll-like receptor (TLR)-mediated innate immune and inflammatory responses contribute to cerebral I/R injury 2–4. TLR-mediated signalling pathways predominately activate NF-κB which is a critical transcription factor regulating gene expression involved in innate and inflammatory responses 5. Recent evidence suggests that TLRs may be important targets for development of new treatment approaches for cerebral I/R injury 6–10. For example, TLR4-deficient mice showed decreased injury following cerebral I/R 6,10. TLR2 has also been reported to play a role in focal cerebral ischaemic injury 10,11. In addition, administration of CpG-ODN, a TLR9 ligand reduces cerebral I/R injury 12,13.

Toll-like receptor 3 is located in intracellular endosomes and recognizes double-stranded RNA (dsRNA), resulting in induction of antiviral immune responses 14. Polyinosinic-polycytidylic acid [Poly (I:C)], a synthetic analogue of dsRNA, stimulates TLR3-mediated responses 14. TLR3 also recognizes by-products from apoptotic and necrotic cells 15. TLR3-mediated signalling predominantly activates IRF3 to stimulate type I interferon (IFN) production 5,16. Recently, Packard and Gesuete *et al*. reported that Poly (I:C)-induced preconditioning decreased cerebral I/R injury 17,18. Pan *et al*. reported that pre-treatment of mice with Poly (I:C) attenuated neurological deficits and reduced infarct volume following cerebral I/R injury 19. Cui *et al*. have shown that transient global cerebral ischaemia increased expression of TLR3, interferon regulatory factor and interferon beta in the hippocampus 20. Collectively, the published data suggest that Poly (I:C) pre-treatment attenuates cerebral I/R injury *via* a preconditioning-dependent mechanism 17,18. However, whether the role of TLR3 in Poly (I:C)-induced protection against cerebral I/R injury has not been investigated.

Microglial cells are the resident macrophages in the central nervous system and they play a critical role in the induction of innate immune and inflammatory responses 21. Increasing evidence suggests that I/R-activated microglial cells induce and release pro-inflammatory cytokines, leading to neuronal damage and dysfunction 22. Activated microglial cells may scavenge damaged neurons 23 and promote regeneration of damaged neurons by secreting growth factors 24. We have previously reported that cerebral I/R induced activation of microglial cells 25. However, it remains unclear whether Poly (I:C)-induced neuroprotection will involve attenuation of microglial activation following cerebral I/R injury.

In this study, we demonstrated that Poly (I:C) administration significantly attenuates murine cerebral I/R injury and that Poly (I:C)-induced neuroprotection is not mediated through preconditioning mechanisms. Of potentially greater clinical importance, therapeutic administration of poly (I:C) reduces cerebral I/R injury. However, the protection by Poly (I:C) is lost in TLR3 knockout mice. We also demonstrated that Poly (I:C) induces co-association between TLR3 and Fas, resulting in preventing I/R-induced activation of Fas/FADD-mediated apoptotic signalling.

## Materials and methods

### Animals

Age- and weight-matched male C57BL/6 mice and TLR3 knockout (TLR3 KO) mice on the C57BL/6 background were obtained from Jackson Laboratory (Indianapolis, IN, USA). The TLR3 KO mice were backcrossed with C57BL/6 for eight interbreeding generations. The mice were maintained in the Division of Laboratory Animal Resources at East Tennessee State University (ETSU). The experiments outlined in this study conform to the Guide for the Care and Use of Laboratory Animals published by the National Institutes of Health (NIH Publication, 8th Edition, 2011). The animal care and experimental protocols were approved by the ETSU Committee on Animal Care.

### Focal cerebral ischaemia/reperfusion

Focal cerebral I/R was induced by occlusion of the middle cerebral artery (MCA) on the left side as described in our previous studies 9,10,13,25,26. Briefly, mice (23–25 g bodyweight) were anaesthetized by 5.0% Isoflurane and anaesthesia was maintained by inhalation of 1.5–2% Isoflurane driven by 100% oxygen flow. Mice were intubated and ventilated with room air using a rodent ventilator at a rate of 110 breaths per min. with a total delivered volume of 0.5 ml. Body temperature was regulated at 37.0°C by surface water heating. Following the skin incision, the left common carotid artery (CCA), the external carotid artery (ECA) and the internal carotid artery (ICA) were carefully exposed. Microvascular aneurysm clips were applied to the left CCA and the ICA. A coated 6-0 filament (6023PK, Doccol Corp., Sharon, MA, USA) was introduced into an arteriotomy hole, fed distally into the ICA. After the ICA clamp was removed, the filament was advanced 11 mm from the carotid bifurcation, and focal cerebral ischaemia was started. After ischaemia for 60 min., the filament and the CCA clamp were gently removed (reperfusion starts). The collar suture at the base of the ECA stump was tightened. The skin was closed, anaesthesia discontinued, and the animal allowed to recover in pre-warmed cages. Control mice underwent a neck dissection and coagulation of the ECA, but no occlusion of the MCA.

### Measurement of cerebral blood flow

Successful occlusion of the MCA was verified and recorded by laser Doppler flowmetry (Model PeriFlux system 5000; Perimed, Stockholm, Sweden) as described previously 13,25. Briefly under anaesthesia, a midline incision of the head was made and a probe holder was attached to the skull with super-crazy glue at 6 mm lateral and 1 mm posterior of bregma. A laser Doppler probe was connected to the probe holder and regional cerebral blood flow (rCBF) was monitored and recorded. The data were collected continuously, stored in a computer, and analysed using the Perimed data acquisition and analysis system. Regional CBF was expressed as a percentage of pre-ischaemic baseline values.

### Experimental design

To evaluate the effect of Poly (I:C) on focal cerebral I/R injury, we employed a non-preconditioning regimen. Poly (I:C) (Catalog Cod: tlrl-picw, InvivoGen, San Diego, CA, USA) was dissolved in sterile endotoxin-free 0.9% NaCl and injected intraperitoneally (i.p., 10 μg/25 g bodyweight, *n* + 8) 1 hr prior to cerebral ischaemia (60 min.) followed by reperfusion for 24 hrs.

To investigate the therapeutic effect of Poly (I:C) on focal cerebral I/R injury, Poly (I:C) (i.p., 10 μg/25 g bodyweight, *n* + 8) was administered by intravascular injection 30 min. after the beginning of cerebral ischaemia. Focal cerebral ischaemia was continued for an additional 30 min. followed by reperfusion for 24 hrs.

To examine the role of TLR3 in Poly (I:C)-induced protection against cerebral I/R injury, TLR3 KO mice (*n* + 7/group) were treated with or without Poly (I:C) (10 μg/25 g bodyweight) 1 hr before the mice were subjected to focal cerebral ischaemia (60 min.) followed by reperfusion (24 hrs). The infarct size for all experiments was determined by triphenyltetrazolium chloride (TTC) staining as described below 9,10,13,25,26.

### Measurement of infarct volume

The infarct volume was determined as described previously 9,10,13,25,26. After completion of reperfusion, mice were killed and perfused with ice cold PBS *via* the ascending aorta. Brains were removed and sectioned coronally into 2-mm-thick slices. The slices were stained with 2% TTC solution at 37°C for 15 min. followed by fixation with 10% formalin neutral buffer solution (pH 7.4). The infarct areas were traced and quantified with an image-analysis system. Unstained areas (pale colour) were defined as ischaemic lesions. The areas of infarction and the areas of both hemispheres were calculated for each brain slice. An oedema index was calculated by dividing the total volume of the left hemisphere by the total volume of the right hemisphere. The actual infarct volume adjusted for oedema was calculated by dividing the infarct volume by the oedema index 9,10,13,25,26. Infarct volumes are expressed as a percentage of the total brain volume ± SEM.

### Evaluation of neuronal damage in the hippocampal formation

Neuronal damage in brain sections were determined by Nissl's method as described previously 9,10,13,25,26. Paraffin sections cut in the coronal plane at ∼1.5 mm behind the bregma with a thickness of 7 μm were deparaffinized and then stained with 0.1% cresyl violet for 2 min. The sections were evaluated using light microscopy by a neuropathologist.

### Immunohistochemistry double fluorescent staining

Double fluorescent staining was performed to examine caspase-3 activity in microglial cells following cerebral I/R as described previously 9,13. Briefly, brain tissues were immersion-fixed in 4% buffered paraformaldehyde, embedded in paraffin, cut at 7 μm, and stained with a specific anti-cleaved caspase-3 antibody which was labelled with FITC. After washing, the sections were incubated with anti-ionized calcium-binding adapter molecule 1 (IBA1; Santa Cruz Biotechnology Inc., Santa Cruz, CA, USA) at 25°C for 1 hr to stain activated microglial cells. After washing, the sections were incubated with Texas Red conjugated anti-goat antibodies (sc-2783; Santa Cruz, Santa Cruz, CA, USA) for 1 hr at 25°C. The sections were then incubated with DAPI for staining the nucleus. The sections were covered with fluorescence mounting medium (Vector Labs, Burlingame, CA, USA). The images were viewed on an EVOS-fI digital inverted fluorescent microcopy (Advanced Microscopy Group, Bothell, WA, USA). Fields of cortex were randomly examined using a defined rectangular field area for analysis of microglia activation.

### *In vitro* experiments

BV2 microglial cells were provided by Dr. Keshvara at Ohio State University and maintained in DMEM supplemented with 5% foetal bovine serum under 5% CO_2_ at 37°C as described previously 13,25. When the cells reached 70–80% confluence, the medium was changed to a hypoxia medium (NaCl 118 mmol, NaH_2_PO_4_ 24 mmol, CaCl_2_ 2.5 mmol, EDTA 0.5 mmol, Sodium l-lactate 20 mmol, KCl 6 mmol, pH 6.2) before the cells were treated with poly (I:C) at a final concentration of 0.1 μg/ml. The cells were then subjected to hypoxia (2 hrs) followed by reoxygenation (12 hrs) 29.

In separate experiments, the cells were treated with poly (I:C) (0.1 μg/ml) for 0, 5, 15, 30 and 60 min. with four replicates at each time-point. The cells were harvested and cellular proteins were isolated for examination of caspase-8 and caspase-3/7 activities by commercially available kits (Promega, Madison, WI, USA) as described previously 27. Cellular proteins were also subjected to immunoprecipitation with a specific antibody against Fas followed by immunoblot with specific antibodies against FADD or TLR3.

### Immunoprecipitation

Approximately, 800 μg of cellular proteins were immunoprecipitated with 2 μg of antibody against Fas (Santa Cruz Biotechnology Inc.) for 1 hr at 4°C on a rotator followed by an addition of 20 μl protein A/G-agarose beads (Santa Cruz) as described previously 27,30. The immunoprecipitates were washed three times in lysis washing buffer, suspended in loading buffer, and boiled for 5 min. before the immunoprecipitates were subjected to immunoblot with primary antibodies (anti-TLR3, 1:1000, and anti-FADD, 1:1000, Santa Cruz), respectively, followed by secondary antibody (anti-rabbit and antimouse; Sigma-Aldrich, St. Louis, MO, USA).

### Western blots

Briefly, the cellular proteins were separated by SDS-PAGE and transferred onto Hybond ECL membranes (Amersham Pharmacia, Piscataway, NJ, USA). The ECL membranes were incubated with the appropriate primary antibody [anti-Fas, anti-FasL, anti-FADD, anti-JNK (Santa Cruz Biotechnology Inc.), anti-cleaved caspase-3, anti-caspase-8 (Cell Signaling Technology Inc., Danvers, MA, USA)], respectively, followed by incubation with peroxidase-conjugated secondary antibodies (Cell Signaling Technology Inc., Danvers, MA, USA). The signals were detected with the ECL system (Amersham Pharmacia). To control for lane loading, the same membranes were probed with anti-GAPDH (glyceraldehyde-3-phosphate dehydrogenase; Biodesign, Saco, ME, USA) after being washed with stripping buffer. The signals were quantified using a G: Box gel imaging system (Syngene, Fredrick, MD, USA).

### Caspase-3/7 and caspase-8 activities assay

Caspase-3 and caspase-8 activity in brain tissue was measured using a Caspase-Glo assay kit (Madison, WI, USA) according to the manufacturer's protocol as described previously 13.

### Electrophoretic mobility shift assay

Nuclear proteins were isolated from ischaemic cerebral hemispheres as described previously 9,10 and NF-κB binding activity was examined by Light Shift Chemiluminescent electrophoretic mobility shift assay (EMSA) kit (Thermo Scientific, Waltham, MA, USA) according to the instructions of the manufacturer.

### Statistical analysis

Data are presented in figures as mean SEM for experimental groups. Group mean levels were compared with anova (one-way or multifactorial as dictated by the design structure) and the least significant difference procedure (as the *F*-test was statistically significant). Probability levels of 0.05 or smaller were used to indicate statistical significance.

## Results

### The protective effect of poly (I:C) on cerebral I/R injury does not require preconditioning

**Fig 1:**
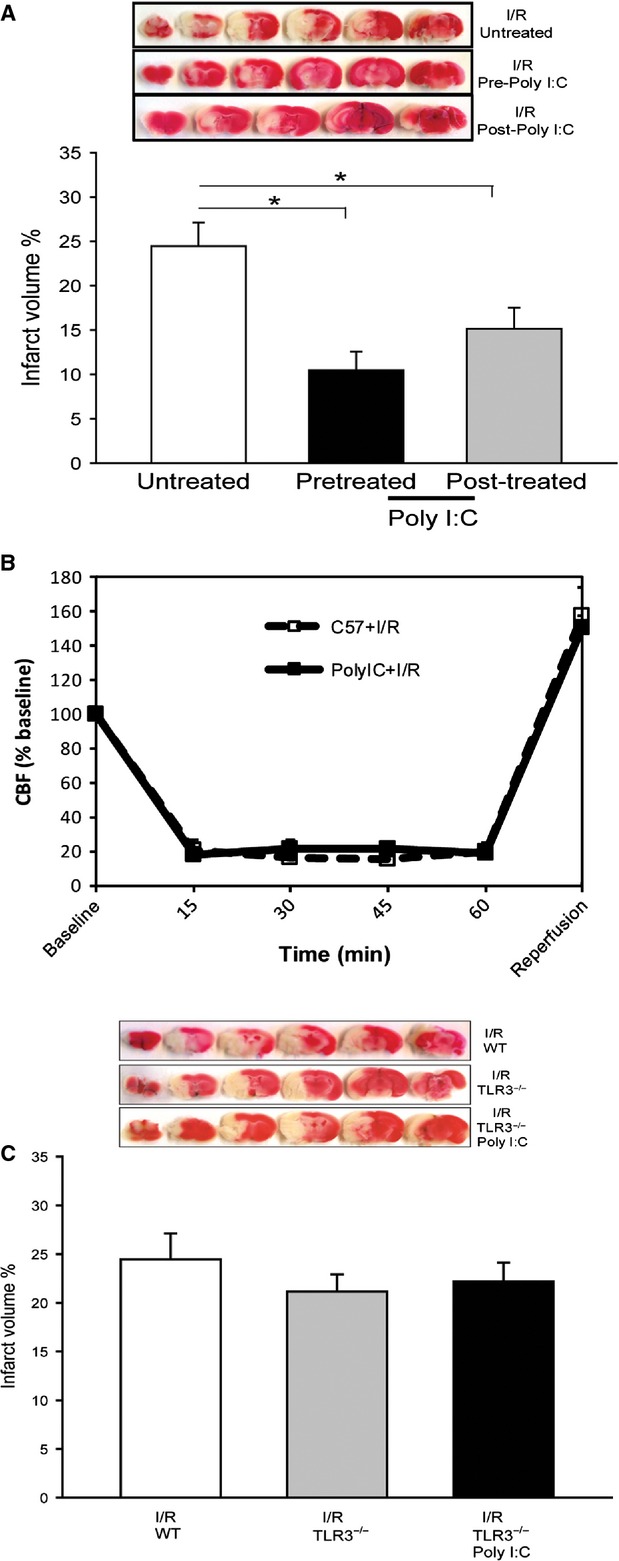
Poly (I:C) administration reduces infarct volume following cerebral I/R. (A) Poly (I:C) (10 μg/25 g bodyweight) was administered to mice 1 hr prior to or 30 min. after cerebral ischaemia. Mice were subjected to cerebral ischaemia (60 min.) followed by reperfusion (24 hrs). Infarct size was examined by TTC staining. Representative image of infarct size from groups are shown on the top of the bar graph. (B) Cerebral blood flow (CBF) measurement before, during and after ischaemia. (C) TLR3 deficiency abolishes the Poly (I:C)-induced protection in cerebral I/R. TLR3 knockout mice were treated with or without Poly (I:C) 1 hr prior to cerebral I/R. *P < 0.05 compared with indicated group. *N* + 7–8/group.

To examine whether Poly (I:C) can induce protection against cerebral I/R injury without preconditioning, we administrated Poly (I:C) to mice 1 hr before the mice were subjected to cerebral ischaemia (60 min.) followed by reperfusion (24 hrs). Figure1A shows that Poly (I:C) administration significantly reduced infarct volume by 57.2% compared with untreated I/R mice. The data indicate that Poly (I:C)-induced neuroprotection occurs rapidly and does not require preconditioning.

### Therapeutic administration of poly (I:C) decreases focal infarct volume following I/R

We also examined the therapeutic effect of Poly (I:C) on cerebral I/R injury. As shown in Figure1A, therapeutic administration of Poly (I:C) 30 min. after the beginning of ischaemia also significantly reduced infarct volume by 34.9% (15.2 ± 2.35 *versus* 24.4 ± 2.67) compared with the untreated I/R group. The data indicate that therapeutic administration of Poly (I:C) during ischaemia decreases I/R-induced brain injury.

### Cerebral blood flow was comparable in control and poly (I:C)-treated I/R mice

It is important to confirm that the effects observed in Poly (I:C)-treated mice were not because of differences in cerebral blood flow after cerebral ischaemia followed by reperfusion. Figure1B shows that cerebral blood flow was significantly reduced by 80%, immediately following occlusion of the MCA. After the occlusion was released, cerebral blood flow returned to slightly above normal levels. There was no significant difference in cerebral blood flow between the untreated cerebral I/R group and the Poly (I:C)-treated group.

### TLR3 deficiency abolished poly (I:C)-induced neuroprotection in cerebral I/R injury

To determine whether TLR3 is required for Poly (I:C)-induced protection against cerebral I/R injury, we treated TLR3 knockout (TLR3^−/−^) mice with Poly (I:C) 1 hr prior to cerebral ischaemia (60 min.) followed by reperfusion (24 hrs). Untreated TLR3^−/−^ mice were also subjected to cerebral I/R. Figure1C shows that the infarct volume in TLR3^−/−^ mice after cerebral I/R was comparable to that in WT I/R mice. Poly (I:C) administration did not reduce cerebral infarction in TLR3^−/−^ mice, indicating that poly (I:C)-induced protection was lost in TLR3^−/−^ mice. The data indicate that TLR3 is essential for mediating the beneficial effect of Poly (I:C) on cerebral I/R injury.

### Poly (I:C) administration attenuated neuronal damage in the hippocampal formation

**Fig 2:**
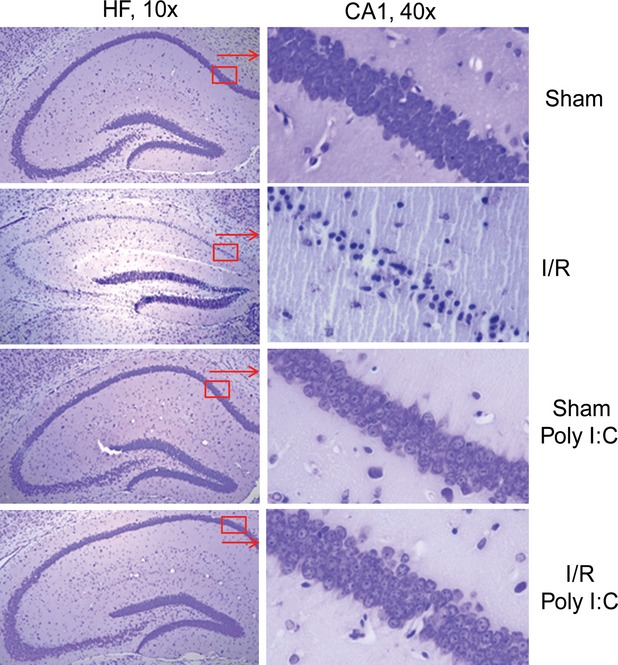
Poly (I:C) treatment attenuates neuronal damage in the HF following cerebral I/R. Mice were treated with or without Poly (I:C) 1 hr prior to cerebral ischaemia (60 min.) followed by reperfusion (24 hrs). Sham surgical operation served as the sham control. Brains were harvested, sectioned and stained with 0.1% cresyl violet (*n* + 4/group). HF indicates hippocampal formation.

We evaluated the effect of Poly (I:C) on neuronal damage following cerebral I/R. Nissl staining showed neuronal damage in the cornu ammonis 1 (CA1) field of the hippocampal formation (HF) characterized by shrunken cell bodies accompanied by shrunken and pyknotic nuclei in the I/R mice (Fig.2). Similar changes were variably expressed in the dentate gyrus (DG). In contrast, the neurons in the CA1 and DG fields in poly (I:C)-treated mice showed less neuronal damage and morphology was preserved.

### Poly (I:C) administration prevents NF-κB binding activity and increases IRF3 phosphorylation in brain tissue following I/R

**Fig 3:**
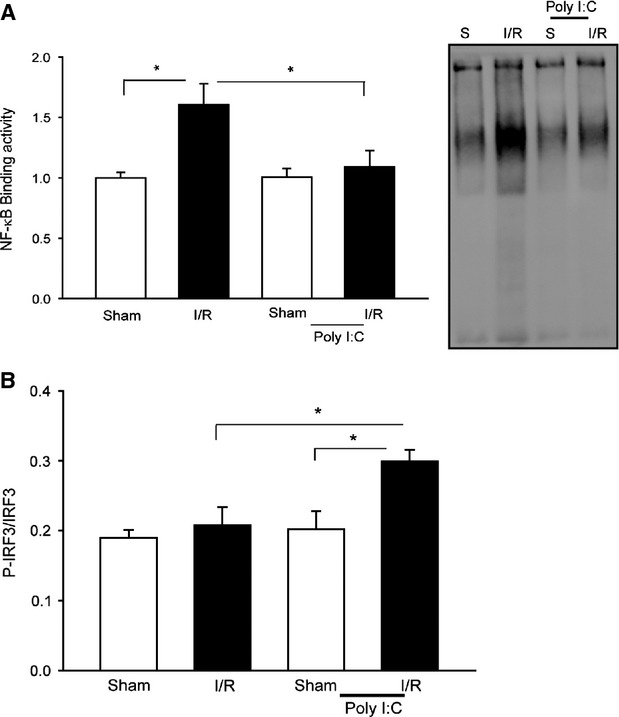
Poly (I:C) administration differentially modulates NF-κB and IRF3 signalling. Mice were treated with or without Poly (I:C) 1 hr prior to cerebral ischaemia (60 min.) followed by reperfusion (6 hrs). Sham surgical operation served as the sham control. The brains were harvested and nuclear and cytoplasmic proteins were isolated. (A) NF-κB binding activity was determined by EMSA. (B) Phospho-IRF3 levels were examined by Western blot with specific antibody. *n* + 5–6/group. *P < 0.05 compared with indicated groups.

NF-κB activation plays an important role in cerebral I/R injury 29,31. Figure3A shows that I/R significantly increased the levels of NF-κB binding activity by 60.7% compared with sham control. In contrast, Poly (I:C) treatment prevented I/R-induced NF-κB binding activity in the brain tissues. TLR3-mediated signalling activates IRF3 which controls IFN expression 5,16. Administration of IFN-β has been demonstrated to induce protection against cerebral I/R injury 30,31. Figure3B shows that Poly (I:C) administration significantly increased IRF3 phosphorylation levels in brain tissue following I/R. In contrast, I/R did not induce IRF3 phosphorylation in brain tissue. The data indicate that Poly (I:C) administration differentially modulates NF-κB and IRF3 signalling pathways. Specifically, Poly (I:C) prevents I/R-induced NF-κB activation and promotes activation of IRF3-mediated signalling.

### Poly (I:C) administration attenuates I/R-induced apoptosis in brain tissues

**Fig 4:**
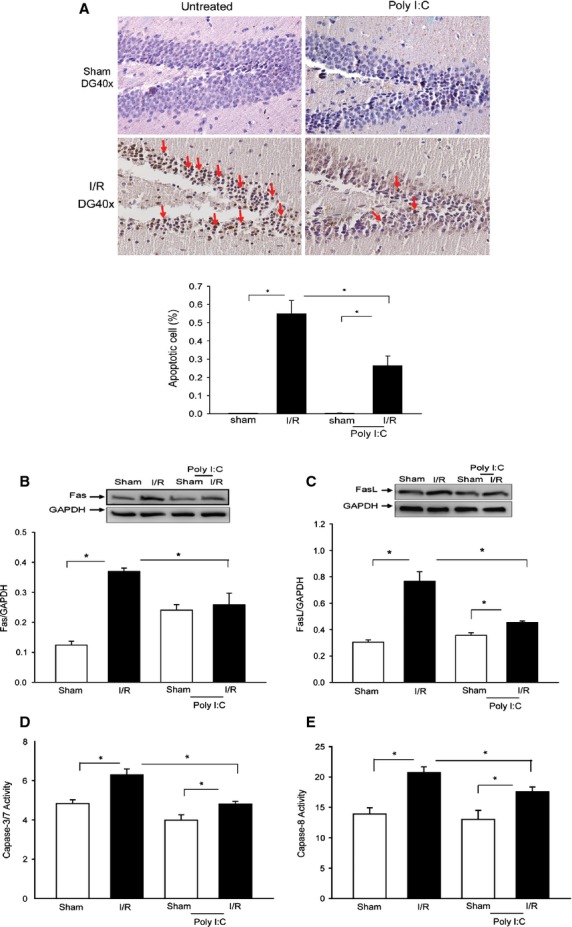
Poly (I:C) treatment attenuates I/R-induced apoptosis in brain tissues. Mice were treated with or without Poly (I:C) 1 hr prior to cerebral ischaemia (60 min.) followed by reperfusion (6 hrs). Sham surgical operation served as the sham control. The brains were harvested and sectioned. Cellular proteins were prepared from the remaining brain tissues. (A) Apoptosis in brain tissue was examined by TUNEL assay. *N* + 4/group. Poly (I:C) treatment decreased the levels of Fas (B) and FasL (C) in brain tissues following cerebral I/R. *n* + 5–6/group. Poly (I:C) attenuates I/R-induced caspase-3/7 (D) and caspase-8 (E) activities which were measured using caspase-3/7 and caspase-8 activity kits. *n* + 4–6/group. *P < 0.05 compared with indicated groups.

Cerebral I/R-induced apoptosis plays a role in brain tissue injury in response to I/R 32. We examined whether administration of Poly (I:C) will attenuate I/R-induced apoptosis in brain tissues. TUNEL staining showed that there were greater numbers of TUNEL-stained positive apoptotic cells in the CA1 field following cerebral I/R compared with the sham control (Fig.4A). However, in Poly (I:C)-treated mice, fewer TUNEL-positive apoptotic cells were observed, indicating that Poly (I:C) administration attenuates cerebral I/R-induced neuronal apoptosis.

### Poly (I:C) administration attenuates Fas and FasL levels in brain tissue following cerebral I/R

The Fas-mediated apoptotic signalling pathway plays an important role in cerebral ischaemic injury 33. We examined the effect of Poly (I:C) administration on Fas and FasL expression in brain tissue following cerebral I/R. Figure4B and C shows that cerebral I/R significantly increased the levels of Fas (B) by 1.97-fold and FasL (C) by 1.50-fold, respectively, when compared with sham control. In Poly (I:C)-treated sham control, the levels of Fas, but not FasL were higher than in untreated sham control. However, I/R-increased levels of Fas and FasL in brain tissues were significantly attenuated by Poly (I:C) treatment. The data indicate that Poly (I:C) attenuated I/R-induced apoptosis through prevention of Fas-mediated apoptotic signalling.

### Poly (I:C) administration attenuates I/R-induced caspase-3/7 and caspase-8 activities in brain tissue

Caspase-3 and -8 activities are the markers for apoptosis 32. Figure4D and E shows that cerebral I/R significantly induced the activities of caspase-3/7 by 30.3% (D) and caspase-8 by 49.1% (E), respectively, in brain tissues, when compared with sham control. In Poly (I:C)-treated mice, cerebral I/R-increased caspase-3 activity was reduced by 23.6% and caspase-8 by 15.3%, respectively, when compared with untreated I/R mice.

### Poly (I:C) inhibits I/R-induced microglial cell activation and attenuates microglial caspase-3 and caspase-8 activity

**Fig 5:**
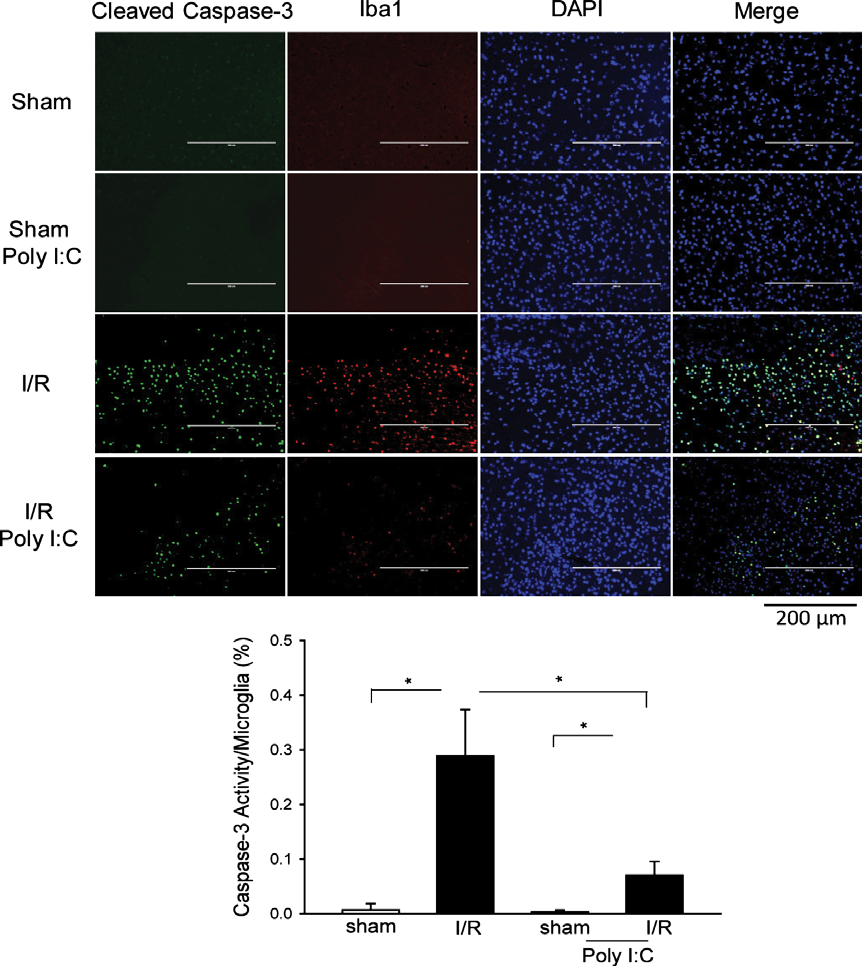
Poly (I:C) administration attenuates I/R-induced caspase-3 activity in microglial cells in the brain tissues. Mice were treated with or without Poly (I:C) 1 hr prior to cerebral ischaemia (60 min.) followed by reperfusion (6 hrs). Sham surgical operation served as the sham control. The brains were harvested and sectioned. Activation of microglia was examined with specific antibody against Iba1 (red). Caspase-3 activity was stained with anti-cleaved caspase-3 antibody (Green). Nuclei were stained with DAPI (blue). Caspase-3 activity (green) in activated microglial cells (red) was a yellow colour (merge). *N* + 4/group. I/R indicate ischaemia/reperfusion. *P < 0.05 compared with indicated groups.

Cerebral I/R also induced caspase-3 activity in microglial cells in the brain tissues 13,25. We examined the effect of Poly (I:C) on caspase-3 activity and microglia activation in brain tissues following cerebral I/R. Immunohistochemistry double fluorescent staining shows that caspase-3 activity (green) and the number of activated microglial cells (red) were low in sham and Poly (I:C)-treated sham groups (Fig.5). However, cerebral I/R resulted in increases in the number of positive caspase-3 staining (green) and the number of activated microglial cells (red) in the brain tissues (Fig.5). In contrast, there were less caspase-3 positive-staining and microglial positive-staining cells in Poly (I:C)-treated mice (Fig.5). The data suggest that Poly (I:C) administration will inhibit I/R-induced microglial cell activation and attenuate I/R-induced microglial caspase-3 activity in brain tissue.

### Poly (I:C) treatment decreased caspase-3 and -8 activities of microglial cells following hypoxia/reoxygenation

**Fig 6:**
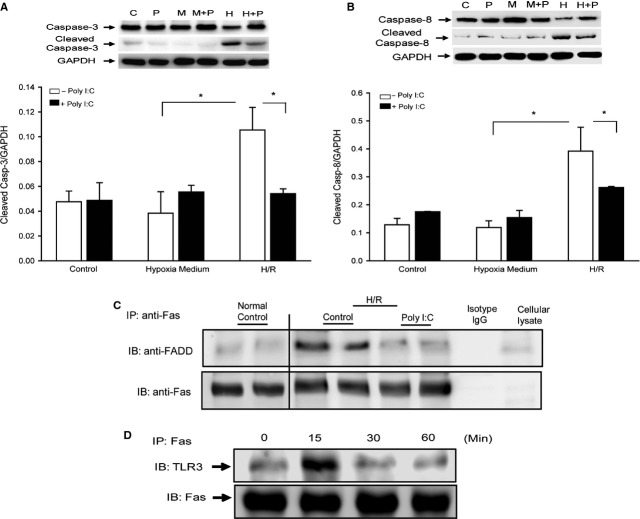
Poly I:C administration prevents hypoxia/reoxygenation (H/R)-induced caspase-3 and caspase-8 activities in microglial cells (Bv2). Microglial cells were treated with and without Poly (I:C) 15 min. before the cells were subjected to hypoxia (2 hrs) followed by reoxygenation (12 hrs). Cellular proteins were isolated for analysis of caspase-3 (A) and caspase-8 (B) activities by Western blot using anti-cleaved caspase-3 and cleaved caspase-8 antibodies, respectively. (C) Poly (I:C) administration prevented H/R-induced interaction of Fas with FADD. Immunoprecipitation (IP) was performed with anti-Fas. The immunoprecipitates were subjected to immunoblot (IB) with anti-FADD and anti-Fas, respectively. (D) Poly (I:C) treatment induced an association between TLR3 and Fas. BV2 cells were treated with or without Poly (I:C) for 0, 15, 30 and 60 min., respectively. Cellular proteins were isolated and immunoprecipitation was performed with specific anti-Fas followed by immunoblot using a specific antibody against TLR3. There were four replicates in each group. Representative blots are shown. M: hypoxia medium; P: Poly (I:C); H/R: Hypoxia/Reoxygenation. *P < 0.05 compared with indicated groups.

*In vivo* data show that Poly (I:C) administration attenuated I/R-induced caspase-3 activity in microglial cells. We performed *in vitro* experiments using microglial cell line BV2 and examined the effect of Poly (I:C) on caspase-3 and caspase-8 activities following hypoxia (2 hrs) followed by reoxygenation (12 hrs). Figure6 shows that hypoxia/reoxygenation (H/R) significantly induced activities of caspase-3 (A) by 118% and caspase-8 (B) by 226%, respectively, compared with control cells that were not subjected to H/R. However, Poly (I:C) treatment prevented H/R-increased caspase-3 and caspase-8 activities in microglial cells.

### Poly (I:C) attenuated hypoxia/reoxygenation-induced co-association between Fas and FADD in microglial cells

*In vivo* data show that Poly (I:C) treatment attenuated cerebral I/R-induced activation of Fas/FasL-mediated apoptotic signalling in brain tissues. We examined whether Poly (I:C) will affect the interaction between Fas and FADD following H/R. Microglial cells were treated with and without Poly (I:C) 15 min. before the cells were subjected to hypoxia (2 hrs) followed by reoxygenation (12 hrs). Control cells were maintained at 5% CO_2_/95% air. Cells were harvested and cellular proteins were isolated for immunoprecipitation with a specific anti-Fas followed by immunoblot with a specific anti-FADD. Figure6C shows that H/R induced an association between Fas and FADD in microglial cells. However, Poly (I:C) treatment prevented H/R-induced interaction between Fas and FADD.

### Poly (I:C) treatment induced a co-association between TLR3 and Fas in cultured microglial cells

We then examined whether there is an association between TLR3 and Fas following Poly (I:C) treatment, thus preventing the interaction of Fas with FADD. Microglial cells were treated with Poly (I:C) for 0, 15, 30 and 60 min., respectively. The cells were harvested and cellular proteins were isolated for immunoprecipitation with a specific anti-Fas antibody followed by immunoblot with a specific anti-TLR3 antibody. Figure6D shows that Poly (I:C) treatment rapidly induced an association between TLR3 and Fas as demonstrated by the presence of TLR3 in the immunoprecipitates with anti-Fas. TLR3/Fas co-association peaked at 15 min. and rapidly decreased thereafter. The data suggest that Poly (I:C) administration induced an association between TLR3 and Fas. TLR3/Fas co-association served to attenuate the H/R-induced Fas with FADD interaction, leading to decreased caspase-8 and caspase-3 activation in microglial cells.

## Discussion

This study showed that Poly (I:C) administration significantly reduced cerebral I/R injury, but the neuroprotective mechanism did not require preconditioning. More significantly, therapeutic administration of Poly (I:C), 30 min. after cerebral ischaemia, also decreased focal infarct volume. However, the protective effect of Poly (I:C) was lost in TLR3-deficient mice, indicating that TLR3 is required for Poly (I:C)-induced protection. Cerebral I/R-induced neuronal apoptosis, microglial activation and microglial caspase-3 activity were significantly attenuated by Poly (I:C) administration. *In vitro* data showed that Poly (I:C) administration induced co-association between TLR3 and Fas in microglial cells, thus preventing the interaction of Fas with FADD and subsequent caspase-3 and -8 activation in microglial cells. We conclude that Poly (I:C)-induced protection against cerebral I/R injury is mediated by TLR3. Mechanistically, Poly (I:C) stimulates TLR3 association with Fas which prevents Fas/FADD-mediated apoptotic signalling.

Recently, published literature indicates that TLR-mediated signalling plays an important role in cerebral I/R injury 6,10,11. TLR4 deficiency 6,8 or TLR2 modulation 9,25 protects the brain from I/R injury. Hyakkoku *et al*. reported that TLR3 deficiency did not induce a neuroprotective effect against cerebral I/R 34, indicating that TLR3 may be required for the induction of protection against cerebral I/R injury. Indeed, we demonstrated in this study that the Poly (I:C)-induced neuroprotective effect was lost in TLR3-deficient mice. Our observation suggests that poly I:C-induced neuroprotection is mediated by a TLR3-dependent mechanism. Packard *et al*. reported that Poly (I:C)-induced preconditioning decreased cerebral injury in response to I/R 17. Pan *et al*. also reported that pre-treatment of mice with Poly (I:C) reduced infarct volume 19. In this study, we observed that Poly (I:C) administration rapidly induces protection against cerebral I/R injury without preconditioning. Of greater importance, therapeutic administration of Poly (I:C) to mice also decreased infarct volume following cerebral I/R injury. Poly (I:C) can be recognized by TLR3 14 which mediates signalling through a TRIF-dependent pathway to stimulate production of IFNs 16. Hua *et al*. and Famakin *et al*. reported that TRIF knockout mice did not show a reduction in cerebral infarction and neurological deficits following cerebral I/R 35,36, indicating that TLR3-mediated TRIF-dependent IFN signalling may serve a protective role in cerebral I/R injury. Indeed, Marsh *et al*. reported that LPS-induced preconditioning decreased cerebral I/R injury through IFN-β production 30. Administration of IFN-β locally protected the brain from ischaemic injury 31. We observed that administration of Poly (I:C) induced IRF3 phosphorylation in brain tissue, indicating that Poly (I:C) administration activates TLR3-mediated TRIF-dependent IRF3 signalling 16.

Cerebral I/R-induced apoptosis plays a role in brain tissue injury in response to I/R 32. In our previous studies, we observed that cerebral I/R induced apoptosis in the CA1 and cortex. This study showed that Poly (I:C) treatment markedly reduced positive apoptotic staining cells in the CA1 field. Poly (I:C) treatment also decreased caspase-3 activity, which is a marker for apoptosis in the cortex. The data indicate that Poly (I:C) administration can attenuate I/R-induced apoptosis. It is well known that activation of Fas by extracellular FasL triggers the recruitment of FADD, which directly activates caspase-8. Activated caspase-8 in turn stimulates caspase-3 activity 37. We observed that Poly (I:C) administration markedly attenuated cerebral I/R-induced increases in Fas and FasL levels as well as caspase-8 and caspase-3 activities in the brain. Activated IRF3 translocates to the nucleus and stimulates the expression of type I interferon genes 16 which are essential for mammalian host defence against viruses. Type I IFNs also have a suppressive effect on immune and inflammatory responses 31,38. In addition, the activated IRF3-mediated IFN pathway is frequently associated with a pro-survival phenotype. Seimon *et al*. 39 have shown that treatment of ER-stressed macrophages with a small dose of LPS induced cell survival through IRF3/IFN signalling. However, blocking TRIF/IRF3 signalling, the cells underwent death by activating JNK-mediated apoptotic signalling. The data indicate that IRF3-mediated signalling promotes cell survival, while suppression of IRF3-mediated signalling leads cell apoptosis. Our data indicate that Poly (I:C) attenuated I/R-induced apoptosis in the brain which could be an important mechanism by which Poly (I:C) induces protection against cerebral I/R injury.

Microglia activation plays an important role in cerebral I/R injury 24. Microglial cells are active sensors and versatile effector cells in pathophysiological brain injury 40. Microglia cells express most TLRs 41, including TLR3 which recognizes Poly (I:C) and double-stranded RNA (dsRNA) 42. We have previously shown cerebral I/R induces caspase-3 and microglia activation in the brain 13,25. Recently, Burguillos *et al*. reported that activation of caspase-8 is associated with microglial activation 43. Activated microglia release substances that cause neuronal injury 21,40. We observed in this study that Poly (I:C) treatment attenuated cerebral I/R-induced caspase-3 activity and microglia activation in the brain, indicating that Poly (I:C) may attenuate microglia activation and apoptosis in response to I/R stimulation.

It has been reported that inhibition of caspase activity in microglial cells protects against neuronal damage in several animal models of brain diseases, including hypoxic or ischaemic stroke and acute bacterial meningitis 43. Inhibition of caspase activity in microglial cells resulted in a neuroprotective effect 44. To further determine the role of Poly (I:C) in attenuation of I/R-induced microglia activation and apoptosis, we performed *in vitro* experiments using the microglial cell line BV2. We observed that Poly (I:C) treatment attenuated H/R-induced caspase-3/7 and caspase-8 activities. Caspase-8 can be activated by Fas-mediated apoptotic signalling through an interaction with FADD 37. We observed that H/R induced an association between Fas and FADD as demonstrated by the presence of FADD in anti-Fas immunoprecipitates. However, the H/R-induced interaction of Fas with FADD was prevented by Poly (I:C) administration. Poly (I:C) can be recognized by TLR3 which expresses on microglial cells 21. To elucidate the mechanism by which Poly (I:C) administration prevented H/R-induced interaction of Fas with FADD, we examined whether Poly (I:C) treatment would promote TLR3 interaction with Fas, thereby, preventing the interaction of Fas with FADD following H/R stimulation. We observed that Poly (I:C) administration induced co-association between TLR3 and Fas as demonstrated by the presence of TLR3 in the immunoprecipitates by anti-Fas. On the basis of these observations, we propose a new neuroprotective mechanism in which Poly (I:C) promotes TLR3 interaction with Fas, thus preventing the interaction of Fas with FADD, thereby, attenuating H/R-induced activation of caspase-8 and caspase-3/7 in microglial cells.

In summary, therapeutic administration of Poly (I:C) significantly reduced cerebral I/R-induced infarct volume *via* a mechanism that does not involve preconditioning. The mechanisms involve attenuation of Fas/FasL-mediated apoptotic signalling. Specifically, Poly (I:C) administration induces a co-association between TLR3 and Fas, thus preventing the interaction of Fas with FADD and the activation of caspase-3 and -8 in microglial cells. The data suggest that Poly (I:C) could be a potential approach for management and treatment of stroke patients.

## References

[b1] Lloyd-Jones D, Adams RJ, Brown TM (2010). Heart disease and stroke statistics–2010 update. Circulation.

[b2] Wang Q, Tang XN, Yenari MA (2007). The inflammatory response in stroke. J Neuroimmunol.

[b3] Stoll G (2002). Inflammatory cytokines in the nervous system: multifunctional mediators in autoimmunity and cerebral ischemia. Rev Neurol.

[b4] del Zoppo GJ (2010). Acute anti-inflammatory approaches to ischemic stroke. Ann N Y Acad Sci.

[b5] Aderem A, Ulevitch RJ (2000). Toll-like receptors in the induction of the innate immune response. Nature.

[b6] Caso J, Pradillo J, Hurtado O (2007). Toll-like receptor 4 is involved in brain damage and inflammation after experimental stroke. Circulation.

[b7] Tang SC, Arumugam TV, Xu X (2007). Pivotal role for neuronal Toll-like receptors in ischemic brain injury and functional deficits. Proc Natl Aad Sci USA.

[b8] Hua F, Ma J, Ha T (2007). Activation of Toll-like receptor 4 signaling contributes to hippocampal neuronal death following global cerebral ischemia/reperfusion. J Neuroimmunol.

[b9] Hua F, Ma J, Ha T (2008). Preconditioning with a TLR2 specific ligand increases resistance to cerebral ischemia/reperfusion injury. J Neuroimmunol.

[b10] Hua F, Ma J, Ha T (2009). Differential roles of TLR2 and TLR4 in acute focal cerebral ischemia/reperfusion injury in mice. Brain Res.

[b11] Lehnardt S, Lehmann S, Kaul D (2007). Toll-like receptor 2 mediates CNS injury in focal cerebral ischemia. J Neuroimmunol.

[b12] Stevens SL, Ciesielski TM, Marsh BJ (2008). Toll-like receptor 9: a new target of ischemic preconditioning in the brain. J Cereb Blood Flow Metab.

[b13] Lu C, Ha T, Wang X (2014). The TLR9 ligand, CpG-ODN, induces protection against cerebral ischemia/reperfusion injury *via* activation of PI3K/Akt signaling. J Am Heart Assoc.

[b14] Matsumoto M, Seya T (2008). TLR3: interferon induction by double-stranded RNA including poly(I:C). Adv Drug Deliv Rev.

[b15] Cavassani KA, Ishii M, Wen H (2008). TLR3 is an endogenous sensor of tissue necrosis during acute inflammatory events. J Exp Med.

[b16] Yamamoto M, Sato S, Hemmi H (2003). Role of adaptor TRIF in the MyD88-independent toll-like receptor signaling pathway. Science.

[b17] Packard AE, Hedges JC, Bahjat FR (2012). Poly-IC preconditioning protects against cerebral and renal ischemia-reperfusion injury. J Cereb Blood Flow Metab.

[b18] Gesuete R, Packard AE, Vartanian KB (2012). Poly-ICLC preconditioning protects the blood-brain barrier against ischemic injury *in vitro* through type 1 interferon signaling. J Neurochem.

[b19] Pan LN, Zhu W, Li C (2012). Toll-like receptor 3 agonist Poly I: C protects against simulated cerebral ischemia *in vitro* and *in vivo*. Acta Pharmacol Sin.

[b20] Cui G, Ye X, Zuo T (2013). Chloroquine pretreatment inhibits toll-like receptor 3 signaling after stroke. Neurosci Lett.

[b21] Olson JK, Miller SD (2004). Microglia initiate central nervous system innate and adaptive immune responses through multiple TLRs. J Immunol.

[b22] Yenari MA, Kauppinen TM, Swanson RA (2010). Microglial activation in stroke: therapeutic targets. Neurotherapeutics.

[b23] Madinier A, Bertrand N, Mossiat C (2009). Microglial involvement in neuroplastic changes following focal brain ischemia in rats. PLoS ONE.

[b24] Lai AY, Todd KG (2006). Microglia in cerebral ischemia: molecular actions and interactions. Can J Physiol Pharmacol.

[b25] Lu C, Liu L, Chen Y (2011). TLR2 ligand induces protection against cerebral ischemia/reperfusion injury *via* activation of phosphoinositide 3-kinase/Akt signaling. J Immunol.

[b26] Lu C, Hua F, Liu L (2010). Scavenger receptor class-A has a central role in cerebral ischemia/reperfusion injury. J Cereb Blood Flow Metab.

[b27] Li C, Ha T, Kelley J (2004). Modulating Toll-like receptor mediated signaling by (1–>3)-β-D-glucan rapidly induces cardioprotection. Cardiovasc Res.

[b28] Ha T, Hu Y, Liu L (2010). TLR2 ligands induce cardioprotection against ischemia/reperfusion injury through a PI3K/Akt-dependent mechanism. Cardiovasc Res.

[b29] Schneider A, Martin-Villalba A, Weih F (1999). NF-κB is activated and promotes cell death in focal cerebral ischemia. Nat Med.

[b30] Marsh B, Stevens SL, Packard AEB (2009). Systemic lipopolysaccharide protects the brain from ischemic injury by reprogramming the response of the brain to stroke: a critical role for IRF3. J Neurosci.

[b31] Veldhuis WB, Derksen JW, Floris S (2003). Interferon-beta blocks infiltration of inflammatory cells and reduces infarct volume after ischemic stroke in the rat. J Cereb Blood Flow Metab.

[b32] Broughton BRS, Reutens DC, Sobey CG (2009). Apoptotic mechanisms after cerebral ischemia. Stroke.

[b33] Rosenbaum DM, Gupta G, D'Amore J (2000). Fas (CD95/APO-1) plays a role in the pathophysiology of focal cerebral ischemia. J Neurosci Res.

[b34] Hyakkoku K, Hamanaka J, Tsuruma K (2010). Toll-like receptor 4 (TLR4), but not TLR3 or TLR9, knock-out mice have meuroprotective effects against focal cerebral ischemia. Neuroscience.

[b35] Hua F, Wang J, Sayeed I (2009). The TRIF-dependent signaling pathway is not required for acute cerebral ischemia/reperfusion injury in mice. Biochem Biophys Res Commun.

[b36] Famakin BM, Mou Y, Ruetzler CA (2011). Disruption of downstream MyD88 or TRIF Toll-like receptor signaling does not protect against cerebral ischemia. Brain Res.

[b37] Chinnaiyan AM, O'Rourke K, Tewari M (1995). FADD, a novel death domain-containing protein, interacts with the death domain of Fas and initiates apoptosis. Cell.

[b38] Reboldi A, Dang EV, McDonald JG (2014). 25-Hydroxycholesterol suppresses interleukin-1-driven inflammation downstream of type I interferon. Science.

[b39] Seimon TA, Obstfeld A, Moore KJ (2006). Combinatorial pattern recognition receptor signaling alters the balance of life and death in macrophages. Proc Natl Aad Sci USA.

[b40] Weinstein JR, Koerner IP, Moller T (2010). Microglia in ischemia brain injury. Future Neurol.

[b41] Jack CS, Arbour N, Manusow J (2005). TLR signaling tailors innate immune responses in human microglia and astrocytes. J Immunol.

[b42] Town T, Jeng D, Alexopoulou L (2006). Microglia recognize double-stranded RNA *via* TLR3. J Immunol.

[b43] Burguillos MA, Dejerborg T, Kavanagh E (2011). Caspase signalling controls microglia activation and neurotoxicity. Nature.

[b44] Braun JS, Novak R, Herzog KH (1999). Neuroprotection by a caspase inhibitor in acute bacterial meningitis. Nat Med.

